# Role of Epigenetic Modulation in Neurodegenerative Diseases: Implications of Phytochemical Interventions

**DOI:** 10.3390/antiox13050606

**Published:** 2024-05-15

**Authors:** Mani Iyer Prasanth, Bhagavathi Sundaram Sivamaruthi, Clerance Su Yee Cheong, Kanika Verma, Tewin Tencomnao, James Michael Brimson, Anchalee Prasansuklab

**Affiliations:** 1 Natural Products for Neuroprotection and Anti-Ageing Research Unit, Chulalongkorn University, Bangkok 10330, Thailand; prasanth.m.iyer@gmail.com (M.I.P.); clerancecheong@gmail.com (C.S.Y.C.); kanika.honey.verma@gmail.com (K.V.); tewin.t@chula.ac.th (T.T.); james.b@chula.ac.th (J.M.B.); 2 Department of Clinical Chemistry, Faculty of Allied Health Sciences, Chulalongkorn University, Bangkok 10330, Thailand; 3 Office of Research Administration, Chiang Mai University, Chiang Mai 50200, Thailand; sivamaruthi.b@cmu.ac.th; 4 Innovation Center for Holistic Health, Nutraceuticals, and Cosmeceuticals, Faculty of Pharmacy, Chiang Mai University, Chiang Mai 50200, Thailand; 5 Research, Innovation and International Affairs, Faculty of Allied Health Sciences, Chulalongkorn University, Bangkok 10330, Thailand; 6 College of Public Health Sciences, Chulalongkorn University, Bangkok 10330, Thailand

**Keywords:** epigenetic modulation, Alzheimer’s disease, Parkinson’s disease, phytochemicals, polyphenols, nutriepigenomics

## Abstract

Epigenetics defines changes in cell function without involving alterations in DNA sequence. Neuroepigenetics bridges neuroscience and epigenetics by regulating gene expression in the nervous system and its impact on brain function. With the increase in research in recent years, it was observed that alterations in the gene expression did not always originate from changes in the genetic sequence, which has led to understanding the role of epigenetics in neurodegenerative diseases (NDDs) including Alzheimer’s disease (AD) and Parkinson’s disease (PD). Epigenetic alterations contribute to the aberrant expression of genes involved in neuroinflammation, protein aggregation, and neuronal death. Natural phytochemicals have shown promise as potential therapeutic agents against NDDs because of their antioxidant, anti-inflammatory, and neuroprotective effects in cellular and animal models. For instance, resveratrol (grapes), curcumin (turmeric), and epigallocatechin gallate (EGCG; green tea) exhibit neuroprotective effects through their influence on DNA methylation patterns, histone acetylation, and non-coding RNA expression profiles. Phytochemicals also aid in slowing disease progression, preserving neuronal function, and enhancing cognitive and motor abilities. The present review focuses on various epigenetic modifications involved in the pathology of NDDs, including AD and PD, gene expression regulation related to epigenetic alterations, and the role of specific polyphenols in influencing epigenetic modifications in AD and PD.

## 1. Introduction

Epigenetics, an intricate field at the crossroads of genetics and environmental influences, is a pivotal avenue for unraveling the complexity inherent in gene regulation and cellular function. Going beyond the traditional focus on the linear sequence of DNA, epigenetics delves into reversible modifications on a cell’s DNA bases or histones, affecting gene expression that transpires without modifying the fundamental genetic code [1,2]. Understanding epigenetic mechanisms is crucial for unraveling the molecular intricacies governing cell differentiation, developmental and biological processes, response to environmental cues, and the manifestation of diverse phenotypes in human health and disease.

**Figure 1:**
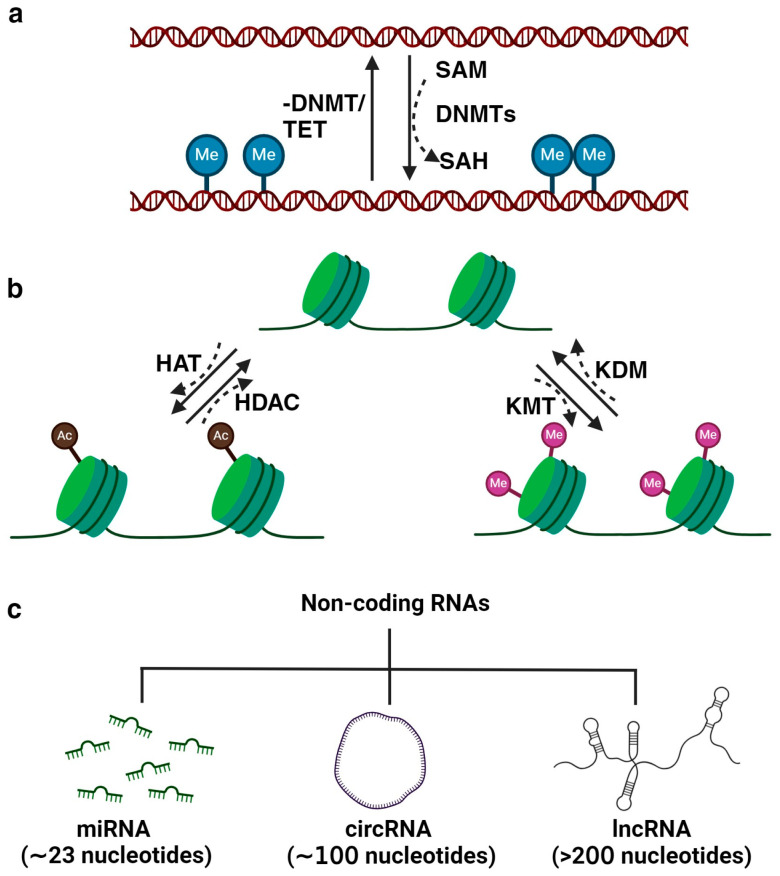
Illustration of essential epigenetic mechanisms governing gene expression by regulating transcriptional activation and repression. (**a**) DNA methylation/demethylation involves adding or removing methyl groups to DNA, respectively, particularly at CpG dinucleotides, influencing gene silencing or activation. (**b**) Histone modifications, including acetylation/deacetylation and methylation/demethylation, modulate chromatin structure, promoting either transcriptional activation or repression. (**c**) Additionally, non-coding RNAs (ncRNAs) such as miRNA, circRNA, and lncRNA participate in fine-tuning gene expression by targeting mRNA stability or acting as molecular decoys.

Three primary mechanisms of epigenetic regulation are illustrated and detailed in Figure 1. DNA methylation is the process of adding a methyl group to a cytosine base in DNA. This modification typically represses gene expression. In mammals, DNA methylation predominantly occurs on cytosines at CpG sites and is facilitated by DNA methyltransferases (DNMTs; DNMT1, DNMT3a, and DNMT3B), which catalyzes the transfer of methyl group from the critical methyl donor S-adenosylmethionine (SAM) [3]. While DNMTs primarily catalyze DNA methylation, the metabolic enzyme nicotinamide N-methyltransferase (NNMT) indirectly influences this process through its regulation of SAM levels [4,5]. NNMT regulates methylation potential within cells by catalyzing the methylation of nicotinamide (NAM) using SAM as a methyl donor. By controlling the availability of SAM, NNMT influences the cellular balance between methyl donor availability and utilization [6]. The interplay between NNMT, NAM, SAM, and cellular metabolism extends to epigenetic regulation. SAM is not only essential for DNA methylation, which is catalyzed by DNMTs, but also for histone methylation, mediated by HMTs [7]. NNMT-mediated depletion of SAM can disrupt these epigenetic modifications, leading to alterations in chromatin structure and gene expression profiles. Through its modulation of intracellular SAM levels, NNMT intricately governs the expression of genes, which involves a reduction in DNA CpG island methylation, leading to a pronounced upregulation of genes [8]. In essence, NNMT’s influence on SAM concentrations serves as a molecular switch, tipping the balance toward heightened gene expression. NNMT has been emerging as a promising therapeutic target in cancer treatment, and several NNMT inhibitors are under study for the therapeutic management of the disease [9,10,11]. DNA demethylation occurs through active processes involving a group of proteins known as ten-eleven translocation methylcytosine dioxygenases (TET). Alternatively, demethylation can happen passively, where 5-methylcytosine (5 mC) is lost during successive rounds of replication without functional DNMT1 [12]. Histone modification is another mechanism of epigenetic regulation. Histones are proteins around which DNA is packaged, forming a chromatin structure. Various chemical modifications can occur on histone proteins, including acetylation, methylation, and phosphorylation. Depending on the specific modification and location, these modifications can promote or inhibit gene expression [13]. The dynamic process of histone acetylation is intricately controlled by various enzymes, namely histone acetyltransferases (HATs) and histone deacetylases (HDACs). HATs (categorized under p300/CBP, GNATs, MYST, and the transcription factor-related family) attach acetyl groups to histones, resulting in a more relaxed chromatin structure that facilitates transcriptional activation [14]. Conversely, HDACs (Class I, II, III, and IV) remove acetyl groups from histones, leading to a more compact chromatin conformation and restricting gene transcription [15]. In addition to HATs and HDACs, there are two other types of enzymes that regulate histone modifications: histone methyltransferases (HMTs), which add methyl groups to lysine or arginine residues, and histone demethylases (HDMs), which act to eliminate them [16]. Non-coding RNA (ncRNA) is an RNA molecule that does not undergo translation into a protein. However, recent evidence has illuminated the significant role of ncRNAs, including microRNAs (miRNAs; ~23 nucleotides), long ncRNAs (lncRNAs; >200 nucleotides), and circular RNAs (circRNAs; ~100 nucleotides) in various biological as well as pathological processes [17].

Epigenetic regulation is crucial for normal development and cellular differentiation. It plays a significant role in diverse biological processes, such as embryonic development, tissue-specific gene expression, and response to environmental stimuli. Epigenetic mechanisms can modify gene expression outcomes, thereby influencing the phenotypic characteristics of the cell [18]. It also has implications for health and disease, as alterations in epigenetic marks can contribute to various conditions, such as cancer, metabolic syndromes, and neurological disorders.

## 2. Neuroepigenetics

The nervous system orchestrates complex functions with unique epigenetic profiles meticulously tailored to specific roles. Due to this intricacy, the transcription machinery faces an arduous challenge in the nervous system, making it highly responsive to epigenetic disturbances [19]. Consequently, the significance of epigenetics in the nervous system is underscored by the occurrence of severe neurodegenerative diseases (NDDs) resulting from mutations in epigenetic genes [20]. The genetic and epigenetic code interplay has been extensively examined in learning and memory. Pioneering studies have confirmed the critical involvement of epigenetic changes in processes related to learning, memory, and synaptic plasticity [21]. It is also evident that disruptions in epigenetic mechanisms not only hinder normal brain functioning but are also linked to various neurological diseases, particularly Alzheimer’s disease (AD) and Parkinson’s disease (PD).

## 3. Epigenetic Changes in the Pathology of Alzheimer’s Disease

Alzheimer’s disease (AD) stands as the predominant type of dementia globally, representing an age-related neurodegenerative condition that significantly impacts memory and cognitive functions. While clinical symptoms typically surface in individuals aged 65 and above, the development of AD spans several decades, with initial pathogenic processes believed to commence several decades before noticeable symptoms emerge. AD is considered a heterogenous disorder with both familial and sporadic forms. Familial AD (FAD) or early onset AD (EOAD) occurs mainly due to mutations in the genes amyloid precursor proteins (*APP*s), *Presenilin-1* and *2* (*PSEN-1*, *PSEN-2*) [22]. Sporadic AD (SAD) or late-onset AD (LOAD) is multifactorial, with heritability as high as 80% [23]. The *APOE ε4* allele was supposed to be the only gene involved in LOAD for several years. However, many other risk genes involved in cellular pathways have also been identified, such as in the maintenance of synaptic plasticity (phosphatidylinositol binding clathrin assembly protein (*PICALM*), bridging integrator 1 *(BIN1*), CD2-associated protein (*CD2AP*)), in immune function (clusterin (*CLU*), complement C3b/C4b receptor 1 (*CR1*), CD33 molecule (*CD33*), ephrin receptor A1 (*EPHA1*)), in lipid metabolism (ATP binding cassette subfamily A member 7 (*ABCA7*)), and cell signaling (a membrane spanning four domains, A4A and A6E (*MS4A4A*, *MS4A6E*)) [24,25,26].

**Figure 2:**
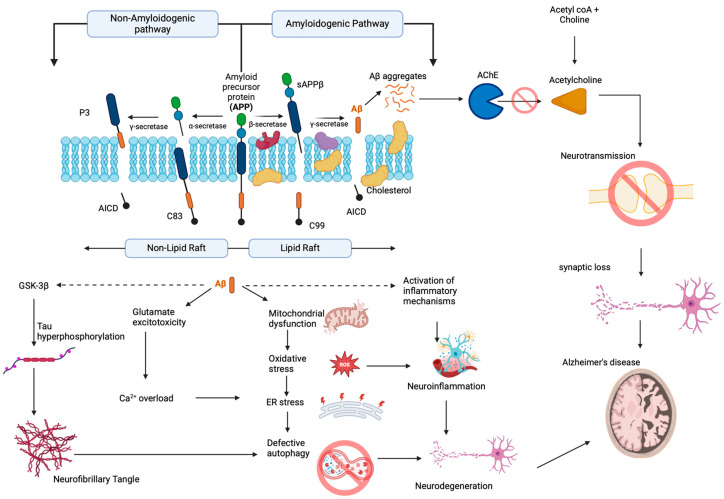
Pathophysiological changes occurring during Alzheimer’s disease (created with BioRender.com).

During AD progression, there occurs a profound loss of forebrain cholinergic neurons coupled with a reduction in acetylcholine in the synaptic cleft due to the aberrant action of cholinesterase enzymes (AChE) leading to cognitive deficits [27,28]. Moreover, there also exists a cross-talk between the cholinergic pathway and amyloid beta pathway (Aβ), and the aberrant disruption of either pathway influences the other [29]. The Aβ peptide is derived from APP, when sequential processing occurs by the enzymes β,γ-secretases instead of non-amyloidogenic processing by α,γ-secretases (Figure 2). The monomeric Aβ undergoes nucleation-dependent polymerization to form oligomer and fibrillar structures and accumulates as senile plaques [30,31,32]. Although under physiological conditions, there exists a balance between Aβ production and elimination through the low-density lipoprotein receptor-related protein (LRP) and enzymatic degradation by neprilysin, the mechanisms are severely impaired in AD patients, contributing to the severity of the disease [33]. Aβ can interact and bind to the receptor for advanced glycation end products (RAGE), which are overexpressed on the membrane of neurons and microglial cells during AD and invoke oxidative stress-mediated degeneration of neurons [34,35,36].

Mounting evidence also suggests that Aβ deposits can invoke neuroinflammation, one of the underlying causes of AD, by activating astrocytes and microglia [37]. Astrocyte and microglial cells activate the nuclear factor kappa B (NF-κB) pathway that subsequently leads to the secretion of several proinflammatory factors, including inducible nitric oxide synthase (iNOS), interleukin-1β (IL-1β), and cyclooxygenase-2 (COX-2), which eventually contributes to neuronal damage and death [38,39,40,41]. In addition to the direct neurotoxic effect, reports show that activated inflammatory cells can help in Aβ accumulation [42]. The secreted cytokines also induce β-secretase expression and enzymatic activity, resulting in increased generation of Aβ [43].

Aβ aggregates show cross-talk between several signaling pathways to aggravate the disease condition. For instance, Aβ oligomers have high affinity toward the receptors including N-methyl-D-aspartate receptors (NMDAR), α-amino-3-hydroxy-5-methyl-4-isoxazole propionic acid receptor (AMPAR), α7 nicotinic acetylcholine receptor (nAChR) and mediate excitotoxicity causing long-term depression (LTD) [44,45,46,47,48]. Aβ oligomers can possibly influence the release of glutamate and inhibit the ability of cells to reuptake the neurotransmitter by the downregulation of glutamate transporters (GLAST-1, GLT-1). These alterations buildup the level of glutamate in the perisynaptic space, resulting in excitotoxicity associated with disruption of Ca^2+^ homeostasis and subsequent degeneration of neurons mediated by ER stress and unfolded protein response (UPR) mechanisms [49,50,51,52]. In addition, as the deposition of Aβ aggregates continues, it leads to the phosphorylation of tau, a microtubule-associated protein located on the axonal compartment that helps in the microtubule stabilization by promoting the assembly of tubulin to microtubules [53]. The aberrant hyperphosphorylation makes the tau protein resist proteolysis by proteases, which in turn impairs the capacity of tau to bind to tubulin, causing deterioration of the microtubule structure, destruction of axonal transport and synaptic metabolism [54]. These changes ultimately result in the disintegration of the cytoskeleton and cause neuronal death. In recent years, researchers have increasingly recognized the pivotal role of epigenetic mechanisms in the progression and course of AD.

### 3.1. DNA Methylation in AD

Mounting evidence indicates that neuroepigenetic modifications that occur naturally during aging due to the involvement of environmental-associated factors act as one of the etiologies for NDDs. Significant correlations were identified linking reduced DNA methylation to the advancement of AD. Elevated levels of NNMT are notably observed in the brains of individuals affected by AD, particularly within the neurons undergoing degeneration, indicating that the upsurge in NNMT expression could be a prevalent characteristic shared among individuals with AD and could lead to hypomethylation, suggesting NNMT-targeted therapy for the treatment [55]. Postmortem brain samples of AD patients have shown a reduction in global DNA methylation in the hippocampal region [56]. In several studies, *APP* and *PSEN1* promoter hypomethylation have been found in AD patients, which is associated with increased amyloid beta peptide (Aβ) [57,58]. Hypomethylation at intron 1 of triggering receptor expressed on myeloid cells 2 (*TREM2*) is observed in AD patients and is associated with increased *TREM2* expression [59]. CREB-regulated transcription coactivator 1 (*CRTC1*) is hypomethylated in the hippocampal region of AD individuals and is correlated with the deposition of p-tau [60]. In late-onset AD (LOAD) individuals, CpGs in *BIN1* (which regulates presynaptic neurotransmitter release) are hypomethylated, leading to increased expression in peripheral blood [61]. A study by Chapuis et al. [62] demonstrated that increased *BIN1* expression induces AD pathogenesis by modulating the tau pathway. Analysis of blood samples from AD patients showed decreased methylation in the mtDNA D-loop region as well as CpG island shores of AD genes *CR1*, *CLU*, and *PICALM* [63,64]. Hypomethylation has also been observed in apolipoprotein E (*APOE*) in the AD brain, which contributes to neural cell dysfunction [65].

The DNA methylation patterns in the human hippocampus revealed hypermethylation in the promoter of the dual-specificity phosphatase 22 (*DUSP22*), which plays a key role in mediating PKA-dependent tau phosphorylation and CREB activation [66]. Hypermethylation of tripartite motif-containing 59 (*TRIM59*) and Kruppel-like factor 14 (*KLF14*) were observed in the peripheral blood obtained from familial AD patients, and bioinformatics analysis revealed that the changes in hypermethylation pattern could induce proapoptotic signaling during AD [67]. Studies indicate that Aβ induced hypermethylation of neprilysin (*NEP*) in the promoter region, thereby suppressing the expression of the enzyme involved in the toxic peptide degradation [68]. Analysis of peripheral blood from AD individuals showed a significantly higher methylation ratio in the brain-derived neurotrophic factor (*BDNF*) promoter compared to the control, with a negative correlation with neuropsychological test subscores indicating the influence in AD manifestation [69,70]. Various studies have reported DNA hypermethylation of ankyrin 1 (*ANK1*) in the postmortem brain of AD individuals, followed by a reduction in expression during the early onset of the disease [71,72,73]. At the internal promoter of protein kinase C zeta (*PRKCZ*; involved in long-term potentiation), DNA hypermethylation was observed in AD individuals and is associated with reduced levels of PKMζ [74].

### 3.2. Histone Modification in AD

Recent epigenome-wide association studies and proteome studies have indicated an increase in histone H3 acetylation (H3K9ac, H3K27ac) in the AD brain, which in turn results in the transcriptional activation of several AD-associated genes to promote neurodegeneration [75,76,77]. A study by Takasu et al. [78] showed that the administration of a HDAC inhibitor in *PSAPP* mice restored gamma oscillation deficits, which is required for regulating synaptic plasticity and cognitive function with concomitant revitalization in the functionality of fast-spiking interneurons. A comparative study in postmortem brain tissue by Nativio et al. [79] showed H4K16ac enrichment associated with neuroplasticity in typically aged individuals and a reduction in AD individuals. Overexpression of HDACs, including HDAC2, 3, and 6, has been related to the repression of genes involved in mitochondrial biogenesis [80], vesicle trafficking [81], and the antioxidant mechanism [82,83,84,85]. Inhibition of class I HDACs in vitro restored Ca^2+^ homeostasis by regulating proteins that control the communication between endoplasmic reticulum and mitochondria [86]. Guan et al. [87] have shown that overexpression of *HDAC2* in mice could cause impaired memory and synaptic plasticity, while knockout reversed the effect. Similarly, *HDAC2* overexpression is also seen in mice models and AD individuals, which could block the expression of neuroplasticity-associated genes [84]. Further, the knockdown of *HDAC2* in APP/PS1 mice increased *BDNF* levels and improved memory [88]. Narayan et al. [89] demonstrated an increase in the level of acetyl H3 and H4 in AD individuals, indicating histone acetylation is not always neuroprotective and aberrant histone modification is involved in neurodegeneration. Further, in support of this notion, RNA-seq analysis of AD postmortem brain revealed an increase in the HATs, which acetylates H3K27 and H3K9 compared to the age-matched control that could worsen Aβ_42_ toxicity besides changes in the methylation pattern of H3K36me1, H3K23me1, and H3K27me3 [77].

Changes in methylation of H2BK108 and H4R55 have been reported in the frontal cortex of the postmortem brain of AD individuals [90]. An increase in histone methylation of H3K9me2 in the *NEP* promoter-1, promoter-2, and HDAC1 was found to be correlated with the downregulation of neprilysin that is required for the degradation of Aβ under hypoxia [91]. Likewise, elevated H3K9me3 is associated with cognitive impairment and reduction of spine plasticity in aged mice [92]. Various studies have shown an aberrant cytoplasmic localization [93] of H3K4me3 as well as an elevated level of H3K4me3 and its catalyzing methyltransferases [94], accompanied by tau hyperphosphorylation. Further, inhibiting specified methyltransferases using inhibitors in AD mice restored synaptic functions and improved memory [94]. Brain tissues from AD mice models and individuals showed an increase of H3K9me2 enrichment at glutamate receptors, impairing synaptic and cognitive functions [95].

### 3.3. Non-Coding RNAs in AD

ncRNAs are involved in biological and pathological processes by interacting with DNA, RNA, and protein [96]. Pioneering studies have confirmed the aberrant expression of ncRNA during NDDs, which alters the transcriptional regulation of genes and modulates cell signaling pathways, further aggravating the disease [97]. For instance, upregulation of miR-206 and miR-613 in transgenic AD mice has been associated with reduced levels of BDNF contributing to AD pathology [98,99]. An increase in the miR-455-3p level in postmortem brains of AD individuals could be positively corroborated with Aβ_1–42_ levels [100,101]. In AD transgenic mice, expression of miR-137 is downregulated and coupled with an increase in calcium voltage-gated channel subunit alpha1 C (*CACNA1C*), and transfection with miR-137 mimics abrogated Aβ_1–42_-mediated tau phosphorylation [102]. Overexpression of miR-137 is also reported to inhibit the neurotoxic behavior of Aβ in neuronal cells by targeting the extracellular signal-regulated kinase 1/2 (ERK1/2) pathway [103]. Overexpression of miR-326 in AD mice improves cognitive function by reducing the level of Aβ and targeting vav guanine nucleotide exchange factor 1 (*VAV1*) to downregulate jun N-terminal kinase (JNK) signaling pathway causing neuroprotection [104]. Upregulation of miR-124 upon transfection with miR-124 mimics decreased the expression of beta-site amyloid precursor protein cleaving enzyme 1 (BACE-1) and prevented the degeneration of neurons against Aβ toxicity [105]. Delivery of miR-16 mimics to the brain of AD mice could effectively reduce BACE-1, tau, and ERK1/2, along with inflammatory and oxidative stress markers [106]. A significant reduction of miR-195 AD individuals carrying a single ApoE ε4 allele, while the overexpression rescued ApoE4-induced cognitive impairment and lysosomal deficits in iPSCs-derived brain cells of ApoE4^+/+^ AD individuals [107]. Pioneering studies have revealed the role of miRNAs in tau protein regulation. Deficiency of miR-132/212 and miR-219 increased tau phosphorylation and aggregation in AD triple transgenic mice and tau *Drosophila* model, respectively [108,109].

Transcriptomic analysis of brain tissues obtained from AD individuals and animal models showed a disrupted pattern in the lncRNA expression compared to control [110,111]. X inactive specific transcript (*XIST*), one of the most extensively studied lncRNAs, was reported to be upregulated in AD animal models. In the AD mice model, lncRNA *XIST* downregulated *NEP* expression and induced Aβ-mediated neuroinflammation, while the knockdown of *XIST* alleviated the effect [112]. A positive and negative correlation was observed between *XIST*-miR-124 and *XIST*-BACE1, respectively, and silencing of *XIST* attenuated BACE1, implicating the pathological influence of XIST in AD [113]. Apart from *XIST*, several lncRNAs, including lncRNA brain cytoplasmic 200 (*BC200*), lncRNA sortilin-related receptor 1 antisense RNA1 (*SORL1-AS1*), lncRNA SOX21 antisense divergent transcript 1 (*SOX21-AS1*), and lncRNA nuclear paraspeckle assembly transcript 1 (*NEAT1*) are also implicated in the pathological process of AD including synaptic impairment, APP processing, and Aβ production and tau phosphorylation [114,115,116,117].

## 4. Epigenetic Changes in the Pathology of Parkinson’s Disease

Parkinson’s disease (PD) is a prominent neurodegenerative condition that affects the motor system, leading to tremors, stiffness, and difficulties in coordination. Unlike AD, PD is not solely age-related, as it can manifest in individuals across a broad age spectrum, although it is more commonly diagnosed in older adults. The hallmark of PD is the progressive loss of dopaminergic neurons in the brain, contributing to the characteristic motor symptoms [118]. While the clinical signs become evident when a significant number of neurons are already compromised, the underlying pathological processes are believed to initiate long before symptoms become apparent.

**Figure 3:**
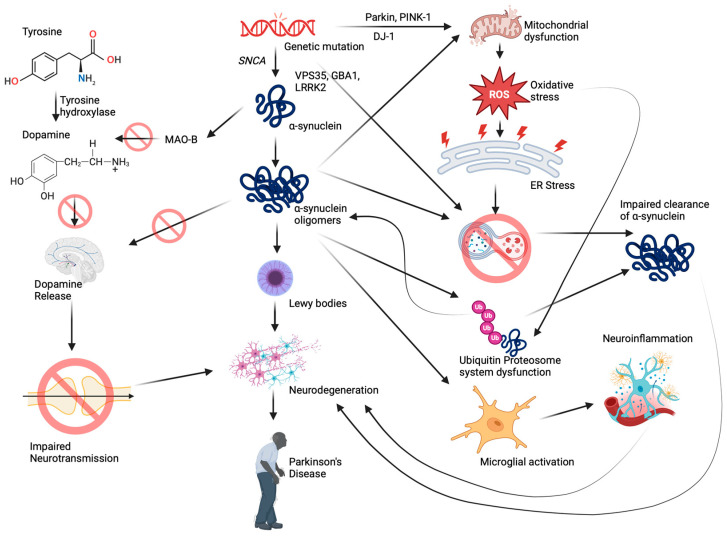
Neurobiological mechanisms involved in the pathogenesis of Parkinson’s disease (created with BioRender.com).

Similar to AD, mutations occurring in some of the important genes play a contributing role in the onset of PD, including synuclein alpha (*SNCA/PARK1*), leucine-rich repeat kinase 2 (*LRRK2*), PTEN-induced kinase 1 antisense RNA (*PINK1*), parkin RBR E3 ubiquitin protein ligase (*PARK2*), Parkinsonism associated deglycase (*PARK7*), vacuolar protein sorting 35 retromer complex component (*VPS35*), glucosylceramidase beta 1 (*GBA1*) that are involved in the regulation of autophagy, unfolded protein response, mitochondrial biogenesis, ubiquitin-proteasome degradation, and survival of dopaminergic neurons [119]. The pathological changes (Figure 3) occur with the accumulation of the oligomeric α-synuclein protein, forming Lewy bodies and resulting in the loss of dopaminergic neurons in the substantia nigra pars compacta [120]. Neuronal loss is further associated with the depletion of the neurotransmitter dopamine, which leads to impaired movement and motor coordination. In addition, there also exists a reduction in the level of serotonin (5-HT) and 5-HT transporter (SERT), affecting serotonergic neurotransmission and further contributing to PD [121]. The mitochondrial enzyme monoamine oxidase B (MAO-B) degrades excess dopamine under physiological conditions [122]. During PD, apart from age-related increase, α-synuclein also induces the expression of MAO-B and stimulates the enzymatic activity by directly binding to them, which in turn makes dopamine unavailable for neurotransmission [123,124].

Genome-wide association studies in PD have highlighted the alterations of several genes and their proteins, which could compromise mitochondrial function, vesicular trafficking, lysosomal functions, and proteostasis [125,126,127]. Studies indicate that there is a deficiency in mitochondrial respiratory chain enzymes in PD individuals [128,129], and accumulation of α-synuclein impairs mitochondrial respiratory chain complexes and promotes oxidative stress [130,131]. Moreover, oligomeric α-synuclein causes oxidation of mitochondrial ATP synthase, resulting in mitochondrial permeability transition pore (mPTP) opening, membrane depolarization, and cell death [132,133]. Under physiological conditions, upon membrane depolarization, translocation of Pink-1 and Parkin to the mitochondria promotes the removal of damaged mitochondria through mitophagy [134,135], whereas pathogenic mutations in *PINK1/PARKIN* cause impaired mitophagy, resulting in the accumulation of defective mitochondria [136,137]. Moreover, extracellular α-synuclein causes Parkin S-nitrosylation, causing auto-ubiquitination and impaired mitophagy [138,139].

Accumulation of misfolded α-synuclein causes impairment of the ubiquitin-proteasome system (UPS) that maintains cellular protein homeostasis. Studies indicate that α-synuclein inhibits the chymotrypsin-like activity of the 20S complex and impairs the degradation system [140,141,142]. In addition, oligomeric α-synuclein interacts with 26S proteasome, inhibiting the proteosomal activity [141,143]. Proteome studies in yeast also show that there is a downregulation of proteins functioning as proteasome subunits upon α-synuclein expression [144]. Mounting evidence also suggests a defect in lysosomal functioning during PD. As the autophagy–lysosome pathway contributes to the clearance of defective/misfolded proteins, lysosomal dysfunction causes the aggregation and accumulation of α-synuclein [145]. Mutations in *GBA*, the gene coding for lysosomal hydrolase enzyme glucocerebrosidase (GCase), have been identified to be related to PD [146]. Knockout of *GBA* in SH-SY5Y cells shows aberrant buildup of enlarged autophagic vesicles and damaged cellular organelles, characteristics of lysosomal dysfunction coupled with deposition of oligomeric α-synuclein [147]. Moreover, reductions of lysosome-associated membrane protein 1 (LAMP-1) and cathepsin D (CatD) have also been reported in the postmortem brain of PD individuals, which acts as markers for lysosomal dysfunction [148]. Mutation or knockout of the *ATP13A2* gene induces CatD deficiency, causing abnormal lysosomal function and preventing the degradation of α-synuclein [149,150]. Moreover, studies indicate that overexpression of α-synuclein downregulates CatD, which could be due to a reduction in mannose 6-phosphate receptor (MPR300) expression that is needed for the transport of cathepsin D from trans-Golgi network (TGN) to endosomes and thereby to lysosomes emphasizing impaired trafficking [151,152]. *A30P* mutation (familial PD mutation) in α-synuclein inhibits the binding of the protein to the vesicle, abrogating the clearance mechanisms [153]. Overexpression of α-synuclein in yeast was reported to cause vesicular trafficking (endocytosis, exocytosis) and endosomal anomalies [154]. Further, α-synuclein blocks endoplasmic reticulum-Golgi traffic, causing neuronal loss [155].

Chronic neuroinflammation is regarded as one of the pathological mechanisms behind PD. The postmortem brain samples of PD individuals show reactive microglia and elevated levels of TNF-α, IL-1β, and IFN-γ [156,157]. Reports show that α-synuclein can instigate inflammatory response, causing further neuronal loss [158,159]. In addition, PD-associated genes including *LRRK2*, *Parkin*, *PINK1*, and *DJ-1* also contribute to neuroinflammation [160,161,162,163]. Recently, researchers have been delving into the role of epigenetic mechanisms in the onset and progression of PD, shedding light on potential avenues for therapeutic interventions.

### 4.1. DNA Methylation in PD

Differential patterns of DNA methylation have also been reported by various studies in the blood and brain samples of PD individuals linked to aberrant gene silencing or reactivation [164,165,166]. For instance, in the postmortem brain of PD individuals, hypomethylation of *SNCA* intron 1 was observed, resulting in increased α-synuclein expression [167]. Hypermethylation was observed in the promoter of *PGC-1α* and reduced mitochondrial biogenesis in the substantia nigra (SN) of PD mice model [168]. Longitudinal genome-wide methylation analysis in blood of PD individuals showed altered methylome patterns on the CpG sites of lamin tail domain-containing 1 (*IFLTD1/LMNTD1*), an intermediate filament protein, and delta-like non-canonical notch ligand 1 (*DLK1*), a transmembrane protein involved in differentiation of multiple cell types [169]. Cai et al. [170] have identified altered expression of clock genes in the leukocytes of PD individuals. Subsequently, methylation in the promoter of clock genes, including cryptochrome circadian regulator 1 (*CRY1*) and neuronal PAS domain protein 2 (*NPAS2*), with significantly decreased methylation frequency of the latter in PD individuals was identified, explaining the disturbed sleeping pattern [171]. Similar to AD, reports suggest an increase in NNMT expression in the degenerating neurons of PD individuals, correlating with hypomethylation [172,173]. Hypomethylation and overexpression of cytochrome P450 family 2 subfamily E member 1 (*CYP2E1*) gene has been reported in the postmortem brain samples of PD individuals, which could be correlated with the increased ROS generation during disease progression [174]. Additionally, SNCA protein expression is downregulated in the brain of *CYP2E1* knockout mice [175]. In PD-like cellular and mice models, hypermethylation of autophagy and beclin 1 regulator 1 (*AMBRA1*) induced dopaminergic neuronal loss via mitophagy [176]. Methylome and transcriptome analysis of the blood of the PD individuals revealed differentially methylated regions, which were mapped near the genes, including nuclear transcription factor Y subunit alpha (*NFYA*), vault RNA 2-1 (*VTRNA2-1*), *cytochrome P450 family 1 subfamily A member 1* (*CYP1A1*) and discoidin domain receptor tyrosine kinase 1 (*DDR1*) [166]. The study conducted by Wu et al. [177] demonstrated an upregulation in the level of cyclin-dependent kinase inhibitor 2A (*CDKN2*) following the overexpression of tet methylcytosine dioxygenase 2 (*TET2*). This increase in *CDKN2* expression led to cell cycle arrest in dopaminergic neurons. Marshall et al. [178] identified 15 differentially methylated cytosine sites within the *TET2* region in PD-connected neurons. Specifically, they observed hypermethylation within an enhancer region of the Tet2 gene body, while the promoter region of *TET2* displayed hypomethylation. Additionally, further investigation revealed that *TET2* inactivation conferred protection against dopaminergic neuronal loss induced by inflammation, suggesting a neuroprotective role for *TET2* loss in PD pathogenesis.

### 4.2. Histone Modification in PD

Genome-wide analysis of histone acetylation in the brain samples of idiopathic PD individuals showed an increase in acetylation of H3K27 similar to that of AD individuals, and prediction analysis indicates a strong link between the altered acetylation and expression of PD-associated genes (synuclein alpha (*SNCA*), microtubule-associated protein tau (*MAPT*), and parkin RBR E3 ubiquitin protein ligase (*PRKN*)) [179]. In *Drosophila melanogaster*, overexpression of α-synuclein induced histone H3 methylation (H3K9me1, H3K9me2) and α-synuclein exposure in differentiated SH-SY5Y cells enhanced H3K9 methylation via *EHMT2* resulting in the upregulation of H3K9me2 [180]. In the postmortem brain of PD patients, enrichment of H3K4me3 was observed in the regulatory region of *SNCA* and was positively correlated with α-synuclein level individuals, and the targeted attenuation reduced α-synuclein levels in the dopaminergic neurons [181]. Midbrain samples from PD individuals showed upregulation of histone acetylation (H2Ak5, H2Bk15, H3k9, and H4k5) compared to control individuals. Additionally, a significant reduction in HDAC1, HDAC2, HDAC6, and SIRT1 was observed in PD mice, indicating increased acetylation attributable to diminished HDACs [182].

### 4.3. Non-Coding RNAs in PD

In the case of PD, overexpression of miR-153 and miR-7 post-transcriptionally controls the *SNCA* expression and regulates the endogenous protein level [183]. Kabaria et al. [184] have reported a reduction in α-synuclein level upon overexpression of miR-34b and miR-34c in neuronal cells, and single nucleotide polymorphism (SNP) in the 3′-UTR of α-synuclein impedes the binding of miRNA’s and makes them resistant resulting in increased expression and aggregation of α-synuclein. Inhibition of miR-96 ameliorated behavioral and motor deficits in PD mice by inhibiting the p38 mitogen-activated protein kinases (MAPK) pathway while increasing calcium voltage-gated channel auxiliary subunit gamma 5 (*CACNG5*) expression [185]. Likewise, inhibition of miR-494-3p attenuated 1-methyl-4-phenylpyridinium (MPP^+^)-induced neurotoxicity by inducing the expression of SIRT-3 [186]. Overexpression of miR-181a, downregulated during PD, significantly reduced autophagy through the downregulation of p38, JNK pathway upon MPP^+^-induced toxicity in SK-N-SH cells [187]. miR-218-5p overexpression alleviated MPTP^+^-induced PD in vivo and suppressed inflammatory response by targeting DEAD-box helicase 41 (*Ddx41*) [188]. The miRNA-sequencing of peripheral blood mononuclear cells (PBMC) from PD individuals showed a reduction in miR-30e-5p, which is positively and negatively correlated with nuclear receptor subfamily 4 group A member 2 (*NURR1*) and NLR family pyrin domain-containing 3 (*NLRP3*) expressions respectively, indicating the role in inflammatory pathology during the disease progression [189]. Likewise, low miR-15b-5p expression induced inflammatory response, apoptosis, and CREB1-induced expression of the same targeted *AXIN2* through the Wnt/β-catenin pathway [190]. MiR-218, a dopaminergic-specific miRNA, promoted dopaminergic differentiation, while the deletion altered dopamine release in mice brain [191].

While evaluating the lncRNA expression in the PBMC of PD individuals, Sarıekiz et al. [192] have observed 13 upregulated (including BTB domain and CNC homolog 1 intronic transcript 2 (*BACH1-IT2*), cAMP-dependent protein kinase inhibitor alpha antisense RNA 1 (*PKIA-AS1*)) and 31 downregulated (including par-3 family cell polarity regulator antisense RNA 1 (*PARD3-AS1*), neighbor of BRCA1 lncRNA 2 (*NBR2*) and MORC family CW-type zinc finger 2 antisense RNA 1 (*MORC2-AS1*)) lncRNAs, which are involved in playing roles in neuroinflammation, intracellular signal transduction and ATPase activity. Microarray analysis in the SN region of the PD individuals showed 87 differentially regulated lncRNAs, among which lncRNA *AL049437* (upregulated) and lncRNA *AK021630* (downregulated) altered significantly. Additionally, in vitro studies showed that *AL049437* and *AK021630* were involved in cell apoptosis and survival, respectively [193]. A significant downregulation in the expression of lncRNA *AK127687* and lncRNA *PINK1-AS1,* along with their mRNAs *LRRK2* and *PINK1* were identified in the SN and cerebellum of PD individuals [194]. The expression of highly regulated lncRNA, *NEAT1* is increased in MPTP-induced PD mice, and knockdown of the same in SH-SY5Y cells exposed to MPP^+^ resulted in a significant decrease in cell apoptosis and increase in dopamine content through the modulation of miR-124/KLF4 axis [195]. In addition, the downregulation of *NEAT1* also repressed NLRP3 inflammasome activation through miR-1301-3p/gap junction protein beta 1 (GJB1) signaling pathway regulation, making it a possible target for PD treatment [196]. The lncRNA distal-less homeobox 6 antisense 1 (*DLX6-AS1*) induced microglial inflammatory response in PD mice models, and silencing alleviated the response through the modulation of miR-223–3p/neuropilin 1 (NRP1) axis [197]. LncRNA HOXA cluster antisense RNA 2 (HOXA-AS2) was considerably upregulated in peripheral blood mononuclear cells of PD individuals, which promoted neuroinflammation and is inversely correlated with peroxisome proliferator-activated receptor-gamma coactivator (PGC-1α) expression [198]. A decline in the level of lncRNA-*T199678* upon α-synuclein toxicity, while overexpression of the same mitigated the effect and protected the dopaminergic neurons by targeting miR-101-3p indicating its essential role in PD [199,200]. A decline in the expression of lncRNA maternally expressed 3 (*MEG3*) and maternally expressed 8 (*MEG8*) has been reported in PD individuals, which is strongly correlated with non-motor symptoms, cognitive deficit, and inflammation, respectively [201,202].

## 5. Nutriepigenomics

Nutrigenomics involves studying how specific nutrients or dietary patterns like vitamins, minerals, and bioactive compounds interact with the epigenome and influence the activity of genes without altering the underlying DNA sequence [203]. Recent evidence underscores the influence of diet on key epigenetic mechanisms, including DNA methylation, histone modifications, and non-coding RNA activity. Adopting a healthy diet not only represents a critical opportunity for reducing disease risk but is especially significant in addressing neurological conditions such as AD and PD. Metabolic and dietary therapies, exemplified by the Mediterranean diet, rich in vegetables, fruits, fish, and oils, have shown potential in decreasing the risk of AD and mild cognitive impairment, emphasizing the significant impact of nutrition on the pathogenesis and progression of neurodegenerative disorders [204].

**Figure 4:**
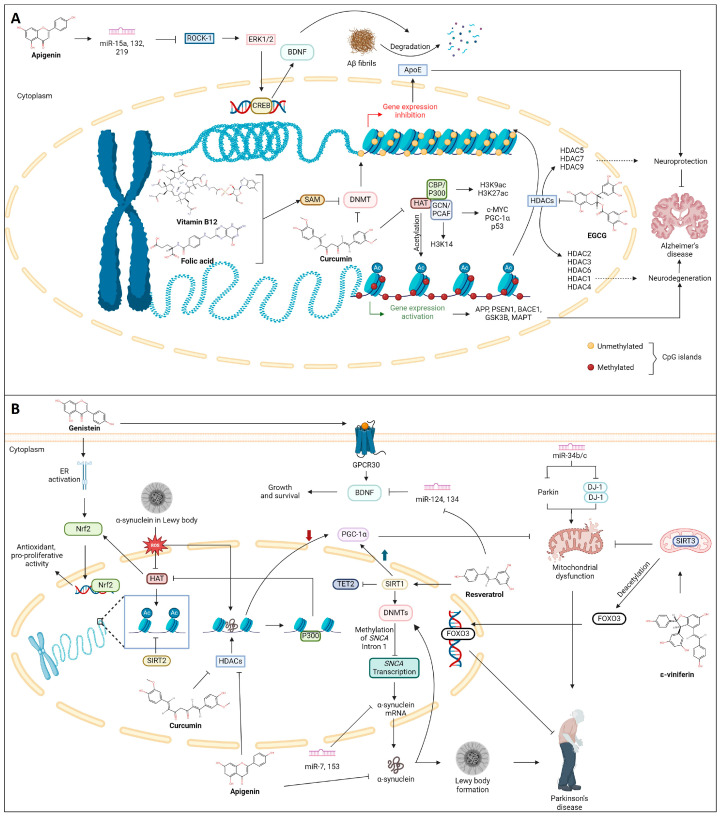
Mechanism of phytochemicals in the regulation of epigenetic modifications in (**A**) Alzheimer’s Disease and (**B**) Parkinson’s Disease (created with BioRender.com).

The sensitivity of the epigenome to diet stems from nutrients and metabolites acting as substrates and cofactors for enzymes involved in DNA, RNA, and histone modifications. This intricate interplay allows diet composition to alter the availability of cofactors, thereby influencing the activity of metabolic enzymes and the binding of gene-regulatory complexes to their substrates [205]. This discovery has transformed our comprehension of the connection between nutrition, health, and disease, opening fresh avenues for exploration and treatment, particularly in NDDs. For instance, the diverse array of components such as vitamins B12, choline, and folate, along with bioactive compounds from tea, garlic, soy, and various natural products, contribute to the modulation of epigenetic mechanisms [206]. As nutriepigenomics sheds light on how dietary factors influence the epigenome, it becomes evident that certain bioactive compounds, such as polyphenols, possess the potential to harness these epigenetic mechanisms for therapeutic benefits, allowing the introduction of the term “epigenetic diet” [207]. Various polyphenols like curcumin, resveratrol, and catechins are noted for their ability to influence NF-κB expression and chromatin remodeling by regulating the activity of HDACs and DNMTs [208]. In this context, the following section delves into the specific polyphenols that have demonstrated promise in influencing epigenetic modifications in AD and PD, primarily during in vitro and in vivo studies, paving the way for a comprehensive exploration of their potential in neuroprotection and disease management. Furthermore, a schematic representation of the influence of phytochemicals in various epigenetic modulations is shown in Figure 4 and listed in Table 1.

## 6. Role of Phytochemicals on the Epigenetic Modification in Combating AD and PD

### 6.1. Tea Polyphenols

The primary tea polyphenols, including catechin, epicatechin, and (−)-epigallocatechin-3-gallate (EGCG), have been identified to inhibit DNMT-mediated DNA methylation in a dose-dependent manner leading to demethylation and gene reactivation [209]. Among these, EGCG, the most potent polyphenol found in tea, exerts its effect on DNA methylation directly or indirectly. It forms hydrogen bonds within the active site of DNMT, directly inhibiting its activity. Additionally, EGCG contributes to histone post-translational modifications by inhibiting HMTs [210]. In the context of tumor-related gene expression regulation, EGCG acts as an inhibitor of HDAC, as demonstrated in neuronal cells, and downregulates the expression of APP [211]. Further, EGCG in AD mice models promoted NEP expression in M146L cells while reducing Aβ level, which correlated with the compound’s inhibitory effect against HDAC1 [212]. It has also been speculated that EGCG, in addition to acting as an inhibitor, also attenuates the competition of HDAC1 with the APP intracellular domain (AICD) for binding to the *NEP* promoter, thereby enhancing neprilysin enzyme induction while decreasing APP and sAPPα levels [239,240].

### 6.2. Resveratrol

The epigenetic mechanisms of resveratrol, which is primarily found in grapes, mulberries, cranberries, and peanuts, occur through histone modifications. Resveratrol exhibits its neuroprotective effect and improves cognitive functions by activating the deacetylase enzyme SIRT1 [241,242]. It has also been reported to inhibit DNMT activity [208]. A study conducted in retinal pigment epithelial cells revealed that resveratrol prevented the decline in expression of DNMT1, DNMT3A, DNMT3B, and SIRT1 triggered by oxidative or inflammatory stimuli [243,244]. Activating endogenous SIRT1 by resveratrol triggers *CDKN2A* DNA hypermethylation by reducing TET2 protein levels. This reduction alleviates the inhibitory influence on cyclin-dependent kinase 4 (CDK4) and promotes the upregulation of pRb, fostering cell proliferation and growth. Correspondingly, analogous outcomes are noted upon inhibiting endogenous TET2 enzyme activity using a TET2 inhibitor, thus presenting a promising target for PD therapy [213].

Additionally, resveratrol may play a pivotal role in regulating the expression of miRNAs, which have implications in neurodegenerative diseases [245]. Interestingly, in neurodegenerative diseases, there is an observed upregulation of miR-21, miR-125, miR-146, and miR-155. Tili et al. [246] demonstrated that resveratrol mitigates the heightened levels of pro-inflammatory miR-155 induced by LPS by enhancing the expression of miR-663. Furthermore, resveratrol was found to decrease the levels of miR-124 and miR-134, thereby promoting the synthesis of BDNF [247,248]. However, conflicting findings exist, as another study suggested that resveratrol treatment leads to an upregulation of miR-124 expression in T cells within the brain [249]. Epigenetic inheritance has been observed in the F1 and F2 generations of mice upon maternal resveratrol supplementation, which also improved cognitive functions with a significant increase in global DNA methylation as well as modulation of *Dnmt3a/b* and *Tet2* expressions [250]. In a separate investigation, rat mothers receiving resveratrol supplementation during perinatal asphyxia showed promising neuroprotective outcomes in their offspring. This effect was characterized by a decrease in inflammatory markers, including interleukin-1β (IL-1β), tumor necrosis factor-α (TNF-α), and S-100 calcium-binding protein B (S-100B), which were regulated by specific miRNAs such as miR-124, miR-132, miR-134, miR-146, and miR-15a [243,251]. A compound structurally akin to resveratrol, ε-viniferin, has been shown to enhance SIRT3 expression and forkhead box O3 (FOXO3) deacetylation. This resulted in reduced mitochondrial depolarization induced by rotenone, decreased neuronal apoptosis, and restoration of the expression of proteins associated with mitochondrial homeostasis in a PD model using SH-SY5Y cells [214,215]. ε-Viniferin, a compound structurally akin to resveratrol, has enhanced SIRT3 expression and FOXO3 deacetylation, protecting against PD in SH-SY5Y cells [214,215].

### 6.3. Curcumin

The main ingredient in turmeric, curcumin, is used by traditional medicinal practitioners in India and China for its antioxidant, anti-inflammatory, antiangiogenic, and anticancer properties. Epidemiological studies performed in India reported that consumption of curcumin leads to a lower incidence of AD in the population [252]. Curcumin can alleviate neurodegeneration and neuroinflammation through multiple mechanisms, including epigenetic changes [253], proven in different experimental models [254]. Curcumin aids in decreasing the production of Aβ by inhibiting the activation of presenilin 1 [255] apart from inhibiting HAT, thereby decreasing histone H3 and H4 acetylation in brain cancer cells [216] and inhibiting DNMT1 activity by covalently binding to the active site of the enzyme [208,256]. In addition, curcumin has been reported to inhibit NNMT in colorectal cancer cells [257].

Curcumin was reported as a natural selective inhibitor of p300 in HAT. Additionally, it suppressed the expression of PS1 and BACE1 by inhibiting histone H3 acetylation in their promoter regions in N2a/APPswe cells, suggesting its role in epigenetic regulation [217]. Curcumin and related curcuminoid compounds could cross the blood-brain barrier and influence the epigenetic regulation in brain cells [220]. Curcumin was reported to reduce the level of methylation of histone H3 at lysine 27 (H3K27me3) jumonji domain-containing protein 3 (JMJD3) within the promoter region of the BDNF, which led to the decrease in the formation of amyloid aggregates thereby boosting mitochondrial function and limiting the Aβ aggregate accumulation [258]. Histone acetylation of the genes associated with AD plays a crucial role in regulating learning and memory and eventually plays a role in AD pathology. Curcumin was reported to alter gene expression by regulating HATs and HDACs activity [259]. It can selectively inhibit the intrinsic E1A-associated 300-kDa protein p300 (p300) in HATs, thereby suppressing the expression of AD-related genes PS1 and BACE1 by inhibiting H3 acetylation in their promoter regions [217].

Curcumin and its metabolites have been observed to inhibit the catalytic thiol group in DNA methyltransferase DNMT1, thereby reducing global DNA methylation levels [260]. Curcumin analogs have upregulated the activity of the Aβ-degrading enzyme neprilysin with DNA methylation modulation through DNMT1 inhibition [218,219]. Curcumin could also induce neuroprotective properties through Wnt pathway modulation [261] as demethoxycurcumin and bisdemethoxycurcumin, the curcuminoid compounds, induce demethylation of Wnt inhibitory factor-1 (*WIF-1*) promoter region via suppressing the activity of DNMT1, and thereby inhibiting the canonical Wnt pathway [220].

Additionally, the epigenetic mechanisms of curcumin have also been regulated through inflammatory pathways, including sirtuins and nuclear factor erythroid 2–related factor 2 (Nrf2) pathways [254]. Curcumin modulates several miRNAs, including the AD-associated apolipoprotein E gene. It regulates the elevation of miR-128 and miR-9, thereby diminishing levels of phosphorylated tau protein and clearing tau tangles from rat cortical neurons [221]. Altogether, curcumin can influence histone modifications, DNA methylation, and levels of miRNAs to modulate Wnt signaling, amyloid processing, and inflammatory pathways, thereby aiding a novel strategy in AD therapeutics [254]. Liposomal-formulated curcumin was reported to target HDAC, prevent apoptosis, and improve motor deficits in Park 7 (DJ-1)-knockout rat model of PD. The curcumin treatment improved motor behavior and motor impairment, blocked neuronal apoptosis, and stimulated DA neurons in the SN, indicating that the nanotechnology-based epigenetics-driven drug discovery platform toward efficacious therapeutics in PD [222].

Even though curcumin is one of the widely used phytochemicals for its health benefits, its safety level is still debatable. The lesser bioavailability of curcumin makes it difficult for it to extrapolate the effects observed in in vitro studies in clinical trials. It was also reported that higher or irregular doses of curcumin could induce DNA damage and ROS production, inactivate the tumor suppressor protein, affect systemic iron metabolism, and inhibit drug-metabolizing enzymes cytochrome P450, glutathione-S-transferase, and UDP-glucuronosyltransferase which, in turn, can hamper the survival of the host [262].

### 6.4. Folic Acid and Vitamin B12

Impaired homocysteine metabolism and deregulation of critical methylation reactions can lead to phosphorylated tau and APP accumulation in the brain [263]. A lower bioavailability of SAM leads to changes in the expression of APP metabolism-regulating genes, which results in increased production and/or accumulation of Aβ peptide. SAM maintains the appropriate methylation of genes involved in APP processing. In that way, silencing these genes prevents the Aβ formation and accumulation, and altered SAM metabolism has been linked to the onset of AD [225]. Reducing folate and vitamin B12 in neuroblastoma cell lines may also lead to a decrease in SAM levels, thereby increasing PSEN1 and BACE levels and Aβ production [223], which the administration of SAM could restore to medium. The SAM-based inhibition of the progression of Alzheimer-like features has also been observed in vitamin-B-deficient animal models [224].

### 6.5. Apigenin

Apigenin (4′,5,7-trihydroxyflavone) is an abundantly found flavonoid in various herbal plants, fruits (oranges), and vegetables, including thyme, chamomile, basil, oranges, onions, and parsley, to name a few [264]. With a demonstrated ability to modify the epigenetics of various diseases, including neurodegenerative disorders, apigenin attenuates the activity of histone deacetylases (HDACs) and restores microRNA (miRNA) expression. It mitigates neuroinflammation [229,265], thereby improving overall cognitive functions. In a previous in vitro study on a preneoplastic JB6 P+ skin cancer cell line, apigenin was shown to reduce DNA methylation in the Nrf promoter via CpG demethylation while halting the expressions of DNMTs (DNMT1, DNMT3a, and DNMT3b) and HDACs (1–8) epigenetic proteins, which reestablished the expression of Nrf2 and elevated the levels of the NAD(P)H:quinone-oxidoreductase-1 (*NQO1*) gene [266]. The administration of apigenin can alleviate neuroinflammation and oxidative stress-induced neuronal apoptosis in PD and postoperative cognitive dysfunction (POCD) [229,267]. This flavonoid has proven to inhibit both the upregulation of NF-kB gene expression and neuroinflammation in substantia nigra pars compacta (SNpc). It also prevents the release of pro-inflammatory cytokines such as TNF-α, IL-β, IL-6, caspase-1, and the pro-inflammatory inducible nitric oxide synthase (iNOS-1) enzyme in PD, and downregulates the levels of IL-2, IL-4, and IL-10 in POCD [229,267,268]. Furthermore, apigenin treatment reduces the level of α-synuclein while positively regulating the protein expression of tyrosine hydrolase (TH) and dopamine D2 receptor, indicating that apigenin confers neuroprotectant properties that may serve as an alternative therapeutic for neurodegeneration disorders like PD [267,268].

Neurodegenerative disorders often lead to histone acetylation and the disruption of gene expression associated with normal cognitive functions [181,269]. One of the target genes of histone acetylation is BNDF, which plays a role in restoring normal cognitive functions. Recombinant BNDF can improve cognitive activity and enhance the synaptic density of the hippocampus in AD [226]. Alterations in BNDF expression levels will affect histone acetylation [227,228]. Apigenin is reported to be able to activate the acetylation of H3 lysine 9 (H3K9) and H4 lysine 12 (H4K12) at the P4 promoter of *BNDF*, followed by the upregulation of downstream calcium/calmodulin-stimulated protein kinase II (p-CAMK II), cAMP response element-binding protein (p-CREB), and extracellular signal-regulated kinase (p-ERK) signaling [229].

Other than cognitive function, histone acetylation is vital for normal memory function [270]. Changes in the plasticity of chromatin and histone acetylation are commonly correlated with memory impairment in aging. In a previous prostate cancer study, apigenin promotes H3K9 and H4K12 acetylation by attenuating the activity of HDAC and decreasing the protein expressions of HDAC1 and HDAC3 [265]. In parallel with cancer study, apigenin can reinstate acetylation levels of both H3 and H4 through the upregulation of CREB binding protein (CBP) and downregulation of HDAC2, proposing apigenin has a vital role in the regulation of histone acetylation in the hippocampus [270].

Moreover, miRNAs have been correlated with the regulation of memory and neurogenesis. Overexpression of miR-15a, miR-132, and miR-219 can enhance spatial memory and overall cognitive ability in AD mice, as AD brains were shown to have downregulation of miR-15a [230,231]. Rho-associated protein kinase-1 (ROCK-1) is essential in the synthesis of inflammatory cytokines in microglial and release by regulating the microglial activation as well as downregulatory effects on ERK 1/2, CREB, and BNDF [232,233]. One AD-related study has shown that miR-15a suppresses the ROCK-1 gene in hippocampus neurons, reducing abnormal tau hyperphosphorylation and neuronal cell morbidity [234]. The neuroprotective effect of apigenin was identified because it significantly increased the miR-15a gene expression and decreased ROCK-1 expression while reversing the activation of microglial and reducing the ionized calcium-binding adapter protein-1 (Iba-1) in methotrexate-treated rat hippocampus [230]. Notably, apigenin could be a potential treatment candidate for epigenetic therapy of neurodegenerative diseases in the future, such as AD and PD.

### 6.6. Genistein

Genistein (4′,5′,7-trihydroxyisoflavone) belongs to the isoflavone class of flavonoids commonly found in soybean-related products. Extensive research has demonstrated its potent anticancer properties, functioning as an inhibitor of epidermal growth factor receptor tyrosine kinase [271]. Beyond its anticancer effects, genistein has been described as having the ability to alleviate symptoms associated with central nervous system (CNS) disorders, including depression, AD, PD, epilepsy, and dementia [272,273,274]. Genistein exhibits promising blood-brain barrier (BBB) permeability, mimicking neuroprotective effects both in vitro and in vivo through its anti-inflammatory activity [275,276]. The neuroprotective effects of estrogens have been evident in various neuronal cell studies [277]. However, estrogen therapy poses potential risks, such as an increased likelihood of various cancers and harm to patients susceptible to blood clotting [271]. Consequently, ongoing research aims to identify estrogen-like phytoestrogens devoid of toxicities. Genistein, with its structural similarity to estrogen, is recognized as a potent phytoestrogen, acting as an agonist binding to estrogen receptors α and β (ERα and Erβ) [278].

Oxidative stress and neuroinflammation contribute to the demise of nigrostriatal dopaminergic neurons [279,280]. Genistein has been identified as a modifier of inflammatory molecules, improving conditions associated with these processes. Recent findings indicate that genistein exerts a neuroprotective effect in a human neuronal SH-SY5Y cell Parkinson’s disease model through ERs and the Nrf2 channels [235]. Activation of Nrf2 by genistein upregulates heme oxygenase 1 (HMOX1), conferring neuronal protection along with glutathione peroxidase. Moreover, genistein is observed to reduce or reverse mitochondrial oxidative stress-induced damage and neuronal cell death [235].

Furthermore, genistein mitigates elevated malondialdehyde (MDA) content without affecting nitrite content or superoxide dismutase (SOD) activity, indicating its ability to reverse Aβ-induced memory deficits by ameliorating oxidative stress [281]. Research by Li et al. [282] similarly describes genistein’s capacity to reduce cerebral infarction and mitigate neuronal damage and apoptosis in neonatal hypoxic-ischemic brain damage (HIBD) mice. This phytoestrogen achieves these effects by mitigating oxidative stress and neuroinflammation via the activation of the Nrf2/HMOX-1 pathway and attenuation of the NF-κB inflammatory pathway, resulting in a total reduction of pro-inflammatory cytokines such as IL-6, IL-1β, and TNF-α [282,283].

To date, limited reports have explored the influence of genistein on gene expression and memory in the brain via epigenetic regulation. Genistein shares similar effects with 17β-estradiol [278], binding to G protein-coupled receptor 30 (GPR30) and enhancing spatial memory function. It also improves the gene expressions of BDNF, insulin-like growth factor 1 (IGF-1), and miR-132 in the hippocampus tissue of ovariectomized rats [284]. Studies suggest that miR-132 expression is associated with neuronal cell growth, synaptic formation, and angiogenesis [238,285]. miR-132 plays a role in memory and learning cognitive function via downregulation of p250GAP, which controls Rac1-PAK-mediated dendrospinogenesis and restoration of the actin polymerization in the dendritic spine of the perirhinal cortex miR-132 plays a crucial role in memory and learning cognitive function by downregulating p250GAP, controlling Rac1-PAK-mediated dendrospinogenesis, and restoring actin polymerization in the dendritic spine of the perirhinal cortex [236,237,238]. These findings suggest that genistein could influence miRNA expression, potentially halting the progression of neurodegenerative diseases.

## 7. Conclusions and Future Perspectives

The exploration of nutriepigenomics within the context of AD and PD offers a compelling avenue for understanding and potentially mitigating the epigenetic dysregulation that underlies these complex neurological conditions. As we deepen our understanding of the intricate interplay between dietary factors and epigenetic mechanisms, we uncover novel opportunities for therapeutic intervention. Nutriepigenomics highlights the significant impact of diet on the epigenome and emphasizes the potential for personalized dietary strategies to modulate disease progression and improve outcomes for individuals with AD and PD. By identifying specific bioactive compounds found in natural sources, we can target key epigenetic pathways implicated in neurodegeneration, offering the possibility of tailored interventions that address the unique molecular profiles of each patient. In addition, the complementary use of phytochemicals alongside available commercial drugs presents a promising avenue for combinatorial treatment approaches. Moving forward, continued research efforts should focus on elucidating the precise mechanisms through which dietary factors exert their epigenetic effects and exploring the feasibility and efficacy of implementing personalized “epigenetic diets” in clinical practice. Additionally, collaborative endeavors between researchers, clinicians, and nutritionists will be essential for translating scientific insights into tangible therapeutic strategies that benefit patients.

In summary, the convergence of neuroepigenetics and nutriepigenomics provides a compelling framework for advancing therapeutic approaches in NDDs. By harnessing the power of dietary interventions to modulate epigenetic mechanisms, we can envision a future where personalized nutritional strategies play a pivotal role in slowing disease progression, preserving cognitive and motor function, and ultimately improving the quality of life for individuals affected by neurological disorders. The intricate interplay between neuroepigenetics and the therapeutic potential of phytochemicals offers a promising avenue for understanding and managing neurodegenerative diseases. However, most of the studies have been done predominantly in in vitro and in vivo models, which, when studied in clinical trials, showed lower bioavailability of certain phytochemicals, limiting their efficacy in reaching therapeutic concentrations in the brain. Additionally, the variability in phytochemical content among natural sources and batch-to-batch variations in supplements can pose challenges in standardizing dosages for consistent therapeutic outcomes. Moreover, some phytochemicals may exhibit side effects or interactions with other medications, necessitating cautious evaluation and monitoring in clinical settings. These factors highlight the need for further research and refinement in harnessing the full therapeutic potential of phytochemical interventions for neurodegenerative disorders. Therefore, future research endeavors should continue to unravel the intricate mechanisms underlying these interactions and their further effects, ultimately translating scientific insights into tangible clinical benefits for individuals grappling with the burdens of AD, PD, and related neurodegenerative disorders.

## Figures and Tables

**Table 1:** Summary of the phytochemicals’ role in the epigenetic modulation of Alzheimer’s and Parkinson’s diseases.

Polyphenol(s)	Structure(s)	Neurodegenerative Diseases	Epigenetic Target Mechanism(s)	Refs.
Tea polyphenols	Catechin 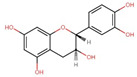 Epicatechin 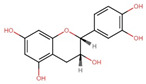 (−)-epigallocatechin-3-gallate (EGCG) 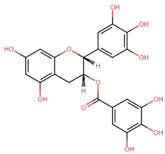	Alzheimer’s disease	- Catechin, epicatechin, and EGCG inhibit DNMT-induced DNA methylation. - EGCG inhibits HMTs, HDAC, ↑ NEP, ↑ neprilysin enzyme induction, ↓ APP, ↓ sAPPα, and ↓ Aβ level.	[209,210,211,212]
Resveratrol	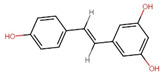	Parkinson’s disease	- Activates SIRT1, ↓ TET2 protein, ↑ *CDKN2A* DNA hypermethylation, ↑ CDK4, ↑ pRb.	[213]
ε-Viniferin	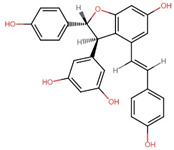	Parkinson’s disease	- Enhances the deacetylation of SIRT3 and FOXO3, ↓ mitochondrial depolarization, ↓ neuronal apoptosis, and restores protein expressions associated with mitochondrial homeostasis.	[214,215]
Curcumin	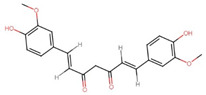	Alzheimer’s disease	- Inhibits activation of PSEN1, inhibits HATs, ↓ H3 and H4 acetylation, ↓ expressions of PS1 and BACE1, leading to ↓ Aβ accumulation. - Suppresses DNMT1 activity, induces deacetylation of *WIF-1* promoter region, ↑ neprilysin activity, and inhibits Wnt signaling. - ↑ miR-128 and miR-9, ↓ phosphorylated tau protein levels, and inhibits Wnt signaling, amyloid processing, and inflammatory pathways.	[216,217,218,219,220,221,222]
		Parkinson’s disease	- Inhibits HDAC, ↓ neuronal apoptosis, stimulates DA neurons in the SN, and improves motor deficits.	
Folic acid and vitamin B12	Folic acid 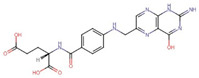 Vitamin B12 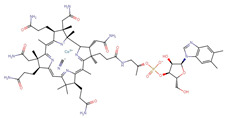	Alzheimer’s disease	- ↓ SAM bioavailability, ↑ PSEN1, ↑ BACE levels, and ↓ Aβ accumulations.	[223,224,225]
Apigenin	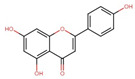	Alzheimer’s disease	- Activates acetylation of H3K9 and H4K12 at the promoter of BNDF, ↑ p-CAMK II, ↑ p-CREB, ↑ p-ERK signaling, improves cognitive activity, and enhances the synaptic density of the hippocampus. - Overexpression of miR-15a, miR-132, and miR-219, ↓ *ROCK-1* gene expression, ↑ ERK 1/2, ↑ CREB, ↑ BNDF, ↓ abnormal tau hyperphosphorylation and neuronal cell morbidity.	[226,227,228,229,230,231,232,233,234]
		Parkinson’s disease	- ↓ NF-kB gene expression, ↓ neuroinflammation, ↓ pro-inflammatory cytokines, ↓ iNOS, ↓ α-synuclein level, ↑ protein expression of TH and D2 receptor.	
Genistein	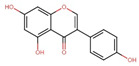	Alzheimer’s disease	- Stimulates GPR30, ↑ spatial memory function, ↑ BNDF, ↑ IGF-1, ↓ p250GAP, ↑ miR-132, and improves memory and learning cognitive function.	[235,236,237,238]
		Parkinson’s disease	- Activates ERs and Nrf2, ↑ HMOX1, ↓ mitochondrial oxidative stress-induced damage, and ↓ neuronal cell death.	

## Data Availability

Not applicable.
